# Evaluating Public Health Resources: What Happens When Funding Disappears?

**DOI:** 10.5888/pcd10.130130

**Published:** 2013-11-14

**Authors:** Ariela M. Freedman, Sarah A. Kuester, Jan Jernigan

**Affiliations:** Author Affiliations: Ariela M. Freedman, Teach For America, Atlanta, Georgia; Jan Jernigan, Centers for Disease Control and Prevention, Atlanta, Georgia.

## Abstract

**Introduction:**

Although various factors affect the sustainability of public health programs, funding levels can influence many aspects of program continuity. Program evaluation in public health typically does not assess the progress of initiatives after discontinuation of funding. The objective of this study was to describe the effect of funding loss following expiration of a 5-year federal grant awarded to state health departments for development of statewide obesity prevention partnerships.

**Methods:**

The study used qualitative methods involving semistructured key informant interviews with state health departments. Data were analyzed using thematic analysis for effect of funding loss on staffing, programs, partnerships, and implementation of state plans.

**Results:**

Many of the programs that continued to run after the grant expired operated at reduced capacity, either reaching fewer people or conducting fewer program activities for the same population. Although many states were able to leverage funding from other sources, this shift in funding source often resulted in priorities changing to meet new funding requirements. Evaluation capacity suffered in all states. Nearly all states reported losing infrastructure and capacity to communicate widely with partners. All states reported a severe or complete loss of their ability to provide training and technical assistance to partners. Despite these reduced capacities, states reported several key resources that facilitated continued work on the state plan.

**Conclusions:**

Decisions regarding continuation of funding are often dependent on budget constraints, evidence of success, and perceived ability to succeed in the future. Evaluating public health funding decisions may help guide development of best practice strategies for supporting long-term program success.

## Introduction

Although various factors affect the sustainability of public health programs (1), funding levels can influence many aspects of program continuity. A recent national report examined government public health funding and proposed recommendations to ensure the continuity of individual public health programs and a sound financial base for the programs (2). A portion of funding for public health systems includes support of workforce infrastructure, information systems, and organizational capacity to deliver services (3). Moulton and colleagues propose a typology of public health finance for use in research, education and training, and performance standards (4); however, program evaluation in public health typically does not assess the progress of initiatives after discontinuation of funding. Understanding how funding changes affect the operation of public health programs can enhance program sustainability when fiscal resources vary over time. The objective of our study was to evaluate the effects of funding discontinuation on federally funded obesity prevention programs in states related to core infrastructure, partnership retention, and the ability to continue state-identified high-priority public health work.

Obesity is a major public health challenge. In 2001, the US Surgeon General called for public health action to prevent and decrease the prevalence of overweight and obesity (5). Since 2000, the Centers for Disease Control and Prevention (CDC) has funded nutrition, physical activity, and obesity (NPAO) programs with state health departments to address obesity. State health departments use CDC NPAO grant funds to 1) develop and implement statewide surveillance systems to define and monitor nutrition and physical activity risk factors and the burden of obesity throughout the state, 2) coordinate activities in state nutrition and physical activity promotion and obesity prevention among partners, 3) develop and foster the implementation of a comprehensive statewide nutrition, physical activity, and obesity plan, and 4) monitor and evaluate the progress and outcomes of the statewide plans.

The CDC NPAO grant program initially funded 6 states and grew to fund 28 states for the grant period of 2003 through 2008. Previous reports have described the accomplishments and progress of NPAO-funded state programs, showing the programs developed strong partnerships across public agencies and private organizations and made policy and environmental changes to create conditions that enable and reinforce healthful lifestyle changes (6–8). State programs were led by a core staff of program managers and physical activity and nutrition coordinators with the support of other disciplines (eg, epidemiologists, evaluators, worksite coordinators).

A previous study of the CDC NPAO grant program used mixed-methods evaluation and described emerging evidence to support assumptions about the centrality of partnerships to states’ success in obesity program development and implementation and related health promotion activities (8). That study also showed that states were able to leverage $2 for every $1 of CDC funding, demonstrating the effectiveness of CDC resources in state-level obesity prevention efforts (8). These findings are similar to those from other obesity prevention infrastructure initiatives that demonstrated the effectiveness of partnerships in leveraging additional funding (9). Other program evaluations describe the critical role of state health departments in supporting and strengthening community-based obesity prevention by providing technical assistance, resources, and evaluation (10).

The CDC NPAO grant provides funding in 5-year increments, after which states reapply for competitive funding through an objective review panel. In 2008, because of resource limitations, CDC funded only 23 states for the 2008 through 2013 grant period; some states received continued funding, some states did not, and some states received funding for the first time. This evaluation examines the effect of discontinued funding on states’ ability to maintain core public health infrastructure, retain partnerships, and conduct state-identified high-priority work.

## Methods

The evaluation used qualitative methods to explore the effect of defunding on state level partnership infrastructure and obesity prevention initiatives. We developed a semistructured interview guide based on interest in understanding the effect of defunding on staffing, programs, partnerships, and state plan implementation. Sample questions from the semistructured interview guide included the following:

- What happened to staffing once CDC funding was no longer available?
- Were nutrition and physical activity programs affected?
- What happened to the partnerships initiated under CDC funding?
- What sustained existing partnerships?
- To what extent is the state plan being implemented?
- What other funding was secured to sustain existing work and partnerships?

Of 13 states that did not receive continued funding, 9 were selected for interviews by using maximum variation sampling related to resiliency, as identified by project officers at CDC. These project officers drew on their experience working with states and their knowledge of what each state accomplished. CDC determined that this evaluation was public health practice and as such did not need human subjects review. No personal identification information was collected. Key informant interviews were conducted by telephone with either a current or former program manager from each state who shared his or her perspective on the outcomes of discontinued funding. Interviews were conducted by CDC staff 18 months following discontinuation of funding to allow sufficient time for the effects of funding loss to become apparent. Two people not previously connected with the program conducted the interviews. One conducted the telephone interviews and one took detailed interview notes. Interview notes were reviewed by both people to ensure accuracy of responses. Notes were subsequently analyzed by theme. To increase interpretive validity of findings, member checks were conducted by sharing results with participating states; states were then asked to provide feedback. Findings were subsequently revised to include participant comments.

## Results

### Effects of discontinued funding on staffing and programs

Loss of funding had a range of effects on staffing. In some state health departments, staffing was significantly reduced, while in others, staffing was completely eliminated. Some states were able to find positions for staff in other areas (which did not allow for continuation of work on NPAO projects), and other states were able to use funding from other sources (eg, Preventive Health Block Grant, tobacco programs, US Department of Agriculture) to pay for the program manager and sometimes one other position for the short-term. In many states, staff members who are expert in nutrition or physical activity left to pursue more stable employment, which resulted in vacancies in these positions that persisted. States that lost nutrition or physical activity specialists reported that the loss of content-area expertise made it difficult to plan or implement specific nutrition or physical activity initiatives. As one respondent explained, “There are no credentialed people on staff, and the lack of expertise is noticeable.”

Although many states were able to shift funds from other sources, this shift often resulted in priorities changing to meet new funding requirements. For some states, this shift resulted in a departure from original priority areas (eg, shifting from NPAO to chronic disease more broadly). Additionally, several state contacts reported needing to spend considerable time each week looking for funding to sustain NPAO work, which detracted from time spent managing existing programs.

Some state health departments had to reorganize their staffing structure to retain positions related to NPAO. For example, one state reported that the loss of funding forced a reorganization to create 4 integrated divisions in chronic disease. Others reported needing to split a position between 2 areas to retain staff (eg, part-time in a stroke prevention program and part-time in worksite wellness). Some states chose to focus on fewer activities in more depth (ie, cut most initiatives completely and direct resources to the few remaining initiatives), while other states chose to spread resources thinly across several initiatives.

Evaluation capacity suffered in all states. Many states had contracted for evaluation services for their programs, but contracts ended when funding stopped. A few states were able to obtain evaluation services on a limited basis through other departments working in chronic disease. Overall, when funding stopped, evaluation was usually the first function to be cut when determining how best to allocate limited remaining resources.

The effect on state programs varied greatly, depending on the type and nature of existing partnerships, the presence of a higher-up NPAO champion (eg, governor), and the ability to shift funds from other areas. For those states without these facilitators, the impact was severe. As one respondent commented, “Programs completely disappeared. They were decimated.”

Some states were able to continue operating existing programs but could not implement programs that were in the planning phases. The inability to implement planned programs was particularly frustrating for partners and staff alike, many of whom had invested significant time in training and capacity building. Furthermore, many of the programs that continued to run were forced to operate under reduced capacity, either reaching fewer people or conducting fewer program activities.

Ultimately, all states reported that loss of funding had a devastating effect on staff morale. While some people lost their jobs completely, others were reassigned to positions outside their area of interest. Most states reported that their staff members with NPAO expertise had been passionate about their work, and both the job loss and reassignment to a different area were demoralizing. As one state health department worker explained, “Another person and I were reassigned into another program we’re not really interested in, but having a job is better than no job.”

All states were frustrated by the inability to proceed with carefully planned initiatives: “It was just unfortunate that we received funding to develop a plan, [and] then we had the rug pulled out from under us as we were ready to implement it [the plan].”

### Effects of discontinued funding on partnerships and state plan

Nearly all states reported losing infrastructure and capacity to communicate widely with partners. One respondent explained, “There is no way for us to collect data on activities – our infrastructure is completely gone. We have completely lost the ability to coordinate, report, and share back any types of communication.” Another respondent reported, “It will be interesting to see what happens with the statistics. . . . Most requests are for stats on school-aged children, and we have no way to provide that information. There is a lack of infrastructure to provide surveillance.”

Overall, there was a strong sense that the state health department served an important function in connecting all NPAO partners, a function that was significantly reduced with the loss of funding. Similarly, all states reported a severe or complete loss of their ability to provide training and technical assistance to partners. In addition to lacking staff time to provide assistance, states also reported lacking money for travel, funding for printing, and expertise to answer content-specific questions.

Many states expressed frustration regarding loss of momentum, stalled plans, or needing to backtrack and re-establish partners and priorities:

Many partners are still engaged, but the intensity around obesity as a central issue is diminishing. . . . Any small amount of money to retain infrastructure would allow for our work to move forward.

Losing funding caused the state to lose ground. Our capacity to provide direction for [NPAO] initiatives was reduced to such a degree that [we] had to re-engage many partners who were already involved . . . and take steps backwards to establish priorities that had been previously clear.

Although some state health departments were able to continue work on the state plan, others were unable to move forward with initiatives because of funding delays. Although many states were able to continue program implementation on some level, nearly all states reported a sense of “treading water” while waiting for the next funding stream to begin. For example, one respondent explained how the continued quest for funding has affected the development of long-range goals: “We have still been able to get a lot done, but there is no ‘long term’ anymore. There is no sense of working towards a vision. There’s no idea how long our jobs will last, so there is no impetus to plan for the future.”

Before funding stopped, state health departments served as coordinators for the majority of NPAO work. Afterward, most states were only able to serve in an advisory role and were no longer able to play a leadership role: “We support the work done by the council, but we aren’t able to lead or direct any of the work. . . . We can’t assist in organizing efforts because we don’t have staff time.”

States reported several key resources that facilitated continued work on the state plan. The most frequently mentioned facilitator was having partners with the same vision and goals:

There has been a sustained effort around the state plan. The old and new partners have stayed very committed.

A new state plan was just published and is being disseminated through various channels. Although this speaks well for the partnership sustainability…. there is little in place for evaluation.

This shared vision, in addition to having a history of working together successfully, kept partners coming to the table despite lack of funding. Additionally, a few states mentioned that having a state leader such as the governor with a passion for NPAO served to maintain momentum (and additional funding) for work on activities related to the state plan.

## Discussion

Our study shows that the loss of state program funding results in decreased capacity to maintain core infrastructure, lead and support partnerships, and conduct state-identified high-priority work to address the public health problem of obesity. Most states experienced loss of staff with content expertise and evaluation capacity. The state programs’ NPAO leadership role was reduced because of staffing losses, resulting in decreased communication and coordination with partners and decreased training and technical assistance. Leadership decisions included choosing areas to decrease program efforts in order to support higher priority areas; however, the capacity to operate in remaining program areas was hampered as well. In some states, the dedication and support of key partners enabled continuation of implementation of segments of the state plan. For some programs, state leaders helped to maintain focus and support for the state plan; in other states variation of funding sources necessitated working on other chronic disease-related program areas with less focus on nutrition and physical activity.

As demonstrated by interviews from this study, partners are often wary of staying engaged in the face of funding that waxes and wanes with each grant season. When a program cannot maintain funding support for partnership infrastructure, it is difficult to sustain the energy and momentum of partnership work. Considering the significant cost of obesity-related diseases now and in the future, states should try to seek diverse sources of funding for large-scale obesity prevention initiatives involving partnership activities. Similarly, funders should focus on the best initiatives even when that may mean not continuing funding of all initiatives.

Previous researchers suggest several ways to enhance program sustainability (11,12). These suggestions include providing sufficient resources and time for grantees to fully develop capacity and yield initial success so they have the ability to obtain funding sources in the future. Another suggested practice is to allocate resources to cover the maintenance costs of existing programs with proven success rather than making investments predominantly in new program directions. In addition to these recommendations, other actions to consider when resources are limited include the following:

1. Evaluating, as a standard practice, grantees that lost funding. One way to show the value of public health is to measure what is lost when resources decrease. For example, several evaluations of tobacco control programs describe the outcomes of reduced funding (13–17). Some of these evaluations identify factors important for sustaining program efforts (18,19). These lessons can inform program funders about managing grants when they face similar reductions.
2. Offering tiered levels of support for grantees at various points in the life of their programs, such as infrastructure and capacity building, initiation and evaluation, and program dissemination. Later support can be tapered so that grantees can seek other sources to maintain and further grow their efforts. These options allow funders to expand with new projects while also transitioning previously successful programs to diversify their own support.
3. Collaborating among program funders to coordinate support for mutually important public health strategies such as what has occurred for obesity through the Convergence Partnership ( http://www.convergencepartnership.org/).
4. Conducting ongoing evaluations of grantees once initiatives are implemented. Suggested frameworks on the sustainability of public health programs include evaluating variables such as client benefits, partnership maintenance, organizational policies and practices, and replication (1). Core domains relevant to grantee evaluation measures have been identified in public health program capacity for sustainability (20). It is also important to track the extent to which grantees diversify their funding sources over time. Continuous evaluations will show to what extent initial program funds can be leveraged to raise new funds to maintain and expand initial projects.

Potential limitations of this study include that CDC, the original funder of the obesity prevention initiatives, conducted the interviews. As such, it is possible that participants may have overemphasized negative effects of funding loss; however, these interviews were conducted approximately a year following funding loss and by staff who had no previous working relationships with the former grantees.

Public health funding decisions must wisely address population needs, especially when resources are limited. Decisions regarding continuation of funding are complex. They are often dependent on budget constraints, evidence of success, and perceived ability to succeed in the future. Evaluating the effect of public health funding decisions may help guide development of best practice strategies for supporting long-term program success. Understanding the effect of funding loss on a program may reveal ways to diminish the effect of that loss. In 2009 no state met the *Healthy People 2010* obesity target of 15%, and the self-reported overall prevalence of obesity among US adults continued to rise (21). Although further efforts in obesity prevention are needed, resource limitations may require funding from multiple sources as a collective effort for long-term support of large-scale health changes (22).
